# A Nano-Liposomal Formulation of Caffeic Acid Phenethyl Ester Modulates Nrf2 and NF-κβ Signaling and Alleviates Experimentally Induced Acute Pancreatitis in a Rat Model

**DOI:** 10.3390/antiox11081536

**Published:** 2022-08-07

**Authors:** Nancy Nabil Shahin, Rehab Nabil Shamma, Iman Saad Ahmed

**Affiliations:** 1 Department of Biochemistry, Faculty of Pharmacy, Cairo University, Cairo 11562, Egypt; 2 Department of Pharmaceutics and Industrial Pharmacy, Faculty of Pharmacy, Cairo University, Cairo 11562, Egypt; 3 Department of Pharmaceutics & Pharmaceutical Technology, College of Pharmacy, University of Sharjah, Sharjah 27272, United Arab Emirates; 4 Research Institute for Medical and Health Sciences, University of Sharjah, Sharjah 27272, United Arab Emirates

**Keywords:** acute pancreatitis, nanoliposomes, ornithine, caffeic acid phenethyl ester, Nrf2, NF-κβ, rats

## Abstract

The currently available management strategies for acute pancreatitis are inadequately effective which calls for exploration of new approaches to treat this condition. Caffeic acid phenethyl ester (CAPE) is a major bioactive constituent of honeybee propolis with promising therapeutic and preventive applications. However, its pharmaceutical potential and clinical use are hindered by its poor water solubility and limited plasma stability. In this study, we aimed to prepare, characterize and evaluate a CAPE-loaded nanoliposomal formulation to improve the efficacy of CAPE for the management of acute pancreatitis. The CAPE-loaded nanoliposomes (CAPE-loaded-NL) were prepared by a thin layer evaporation technique and were optimized using three edge activators. CAPE-loaded-NL were characterized for their vesicle size (VS), zeta potential (ZP), encapsulation efficiency (EE), polydispersity index (PDI), crystalline state and morphology. The protective effect of the optimal CAPE-loaded-NL was evaluated in a rat model of acute pancreatitis induced by administering a single intraperitoneal injection of L-ornithine. Oral pretreatment with CAPE-loaded-NL significantly counteracted ornithine-induced elevation in serum activities of pancreatic digestive enzymes and pancreatic levels of malondialdehyde, nuclear factor kappa B (NF-κB) p65, tumor necrosis factor-alpha, nitrite/nitrate, cleaved caspase-3 and myeloperoxidase activity. Moreover, pretreatment with CAPE-loaded-NL significantly reinstated the ornithine-lowered glutathione reductase activity, glutathione, nuclear factor erythroid 2-related factor 2 (Nrf2), heme oxygenase-1 levels and ATP/ADP ratio, and potentiated the Bcl-2/Bax ratio in pancreatic tissue. CAPE-loaded-NL displayed superior antioxidant, anti-inflammatory and anti-apoptotic effects compared to free CAPE oral suspension and achieved a more potent correction of the derangements in serum amylase and pancreatic myeloperoxidase activities. The histological observations were in line with the biochemical findings. Our results suggest that CAPE-loaded-NL provide a promising interventional approach for acute pancreatitis mainly through the enhancement of the exerted antioxidant, anti-inflammatory and anti-apoptotic effects which may be mediated, at least in part, through modulation of Nrf2 and NF-κβ signaling.

## 1. Introduction

Acute pancreatitis (AP) is a condition of acute inflammation of the pancreas, with varying severity that ranges from mild and self-limiting, to severe and life-threatening, and with varying involvement of regional tissues or organ systems [1]. Mild cases diagnosed with AP usually resolve without complications by supportive measures; however, 20% of the patients develop severe disease accompanied by multiple organ failure resulting in a mortality rate of nearly 30% [2,3].

AP is characterized by interstitial edema, acinar cell necrosis, hemorrhage, and severe inflammation of the pancreas. Patients with AP show high blood and urine levels of pancreatic digestive enzymes, such as amylase and lipase [4]. The pathogenesis of AP has not, thus far, been fully explicated. Yet, a common feature and one of the earliest events in AP pathogenesis is the premature activation of zymogens, mainly trypsinogen, the key enzyme for activating pancreatic zymogens in pancreatic acinar cells [5]. The inappropriate activation of digestive enzymes elicits autodigestion of the gland resulting in pancreatic injury and triggering an inflammatory response. This, in turn, provokes considerable tissue damage and may proceed to a systemic inflammatory response syndrome and multiple organ failure which contribute to the high mortality rate of AP [3].

Oxidative stress is recognized as a major culprit in the pathogenesis of AP with impaired nuclear factor erythroid 2 (NFE2)-related factor 2 (Nrf2) signaling and downstream cascade being implicated as a crucial underlying mechanism [6,7]. Inflammatory events have been encountered in different models of AP, of which activated NF-κβ signaling has been a major trigger. In fact, a key player in pancreatic injury is the intracellular activation of NF-κB, a transcriptional regulator of an array of proinflammatory cytokines and adhesion molecules [6,8,9]. Dysregulated redox signaling and subsequent oxidative stress considerably contribute to the local and systemic inflammatory events encountered in AP [10].

Several animal models have been adopted to study AP among which the ornithine model, induced by intraperitoneal injection of 3 g/kg L-ornithine, represents a simple and non-invasive model of acute necrotizing pancreatitis in rats [11]. The model embodies many biochemical and molecular features that have been detected in other models of AP, including oxidative stress [11,12], IκB protein degradation with subsequent NF-κB activation [9,11], increased pro-inflammatory cytokine production, in addition to dysregulation of pancreatic digestive enzymes [11].

There is no casual treatment in AP and therapy is based on symptomatic treatment. The methods of therapy mainly include aggressive intravenous hydration, early enteral feeding, and intervention in the event of complication [13,14]. The lack of satisfyingly effective treatments for AP raises the demand for further research aiming at affording more efficient interventional modalities.

Caffeic acid phenethyl ester (phenylethyl caffeate, CAPE), a natural polyphenolic flavonoid-like compound, is a major bioactive constituent of honeybee propolis. CAPE has shown several favorable biological and pharmacological properties, including antioxidant, anti-inflammatory, antifungal, antiviral, immunomodulatory and anti-tumor activities [8,15,16,17,18,19]. Importantly, it has not caused any deleterious effects in normal cells [20].

Despite the established beneficial role of CAPE in vitro and in vivo, its efficiency is substantially undermined by its rapid degradation in plasma by the esterase activity present in blood and cells [21]. The rapid clearance and short biological half-life of CAPE result in inadequate bioavailability and weak bioactivity [22]. Moreover, the oral bioavailability of CAPE is limited due to its poor water solubility [23].

Recent research has focused on boosting the efficacy of inadequately bioavailable compounds by nanoencapsulation approaches. Nanocarriers, such as liposomes and nanoparticles, with very small particle size, confer significant effects on the pharmacokinetics and pharmacodynamics of encapsulated drugs by virtue of their greater surface area to volume ratio and are considered as optimal vehicles for targeted drug delivery [23,24].

Liposomes are spherical vesicles consisting of one or more concentric phospholipid bilayers enclosing an aqueous volume [25]. They are composed of biocompatible and biodegradable lipids mimicking biological membranes. Liposomes have been especially attractive to use as nanocarriers for actives due to their flexibility in accommodating different types of actives, ease of preparation, efficient cellular uptake, ability to carry large drug payloads, non-immunogenicity and low toxicity [26]. The use of liposomes has been reported to significantly increase the therapeutic effects of many hydrophilic and lipophilic actives by improving their aqueous solubility, altering their biodistribution profile, increasing their plasma circulation time and protecting them against enzymatic degradation and immunologic inactivation [27].

Although the protective/therapeutic benefits of CAPE have been extensively studied [15,16,17,19], only a few recent studies have reported the inclusion of CAPE in a delivery system [22,28,29].

To our knowledge, this is the first study that aims to develop and characterize a nano-liposomal formulation of CAPE to evaluate its potential in the management of acute pancreatitis following oral administration to rats.

## 2. Materials and Methods

### 2.1. Materials

L-ornithine, caffeic acid phenethyl ester (CAPE), Tween 80 (polysorbate 80), sodium cholate, sodium deoxycholate, L-α-phosphatidylcholine and cholesterol (CH, minimum 99%) were purchased from Sigma-Aldrich Chemical Co. (St. Louis, MO, USA). Ethanol was obtained from El Nasr Pharmaceutical Chemicals (Cairo, Egypt). Other chemicals used were of pure analytical grade.

### 2.2. Preparation of CAPE-Loaded-NL

CAPE-loaded nanoliposomes (CAPE-loaded-NL) were prepared by the thin layer evaporation technique [30]. Briefly, L-α-phosphatidylcholine (PP) and the edge activator (Tween 80, sodium cholate or sodium deoxycholate) were used in the molar ratio 5:1 to which a constant weight of CAPE (10 mg) and cholesterol (25 mg) was added. The ingredients were dissolved in 10 mL ethanol using a bath sonicator (Ultrasonic bath sonicator, Thomas Scientific, Model SH 150-41, Swedesboro, NJ, USA). The resulting solution was then transferred into a 250 mL round bottomed flask and the organic solvent was slowly evaporated under reduced pressure using a rotary evaporator (Rotavapor, Heidolph VV 2000, Heidoph Instruments GmbH and Co., Schwabach, Germany) at 60 °C for 5 min at 120 rpm. A thin dry film was formed on the inner wall of the rotating flask. The dried film was hydrated with 10 mL distilled water by rotation for 1 h at 60 °C to form the nanosuspension. Three edge activators (Tween 80, sodium cholate or sodium deoxycholate) were used in the preparation of the liposomes to investigate the effect of the edge activator on the particle size (PS) and entrapment efficiency (EE) of the prepared liposomes. The edge activator resulting in the formation of the smallest liposomes and an acceptable EE% (>75%) was chosen for the preparation of the optimal nanoliposomes (optimum formulation). Following selection of the edge activator, the optimum formulation was used for further testing and characterization.

The optimum CAPE-loaded-NL suspensions were also mixed with 1% mannitol as cryoprotectant, and the mixtures were frozen at −80 °C for 24 h using Innova U725-G freezer (New Brunswick Scientific, Edison, NJ, USA). The frozen suspensions were then lyophilized in a freeze drier (Freezone Freeze Dryer, Labconco, Kansas City, MO, USA) at −75 °C and 0.003 mbar for 72 h to yield dry powders (lyophilizates). The lyophilizates were then placed in glass bottles, wrapped with aluminum foil, and stored at room temperature in a desiccator until used.

### 2.3. Characterization of CAPE-Loaded-NL

#### 2.3.1. Vesicle Size, Polydispersity Index and Zeta Potential

The mean vesicle size (VS, z-average diameter), polydispersity index (PDI) and zeta potential (ZP) of CAPE-loaded-NL suspensions were determined by photon correlation spectroscopy using Zetasizer Nano ZS-90 Instrument (Malvern Instruments, Malvern, UK). Samples were suitably diluted in distilled water prior to analysis. The PDI is indicative of the size distribution of CAPE-loaded-NL such as small PDI values represent narrow size distributions [31]. The surface charge of CAPE-loaded-NL was also quantified by measuring the ZP. Measurements were performed in triplicate using 90° light scattering angle at 25 °C. The mean value ± SD of three replicates was calculated.

#### 2.3.2. Determination of EE%

The method of determination of the concentration of CAPE was UV-spectrophotometric determination at λmax 330 nm without any calorimetric method. A standard curve between the concentration the UV absorbance of CAPE was developed and was used to calculate the concentration of CAPE in the supernatant.

All assays were performed in triplicate. During the assay of the study samples, the intra-batch precision and accuracy of the analytical procedure were evaluated after replicate analysis (*n* = 6) of control samples spiked at four concentration levels: 2, 4, 6, 8, and 10 mcg/mL. The lower limit of quantification was 2 mcg/mL and a linear response across the full range of concentrations from 2 to 10 mcg/mL (*r*^2^ = 0.999) was obtained. 

To determine the amount of encapsulated CAPE, the NL were separated from the aqueous suspensions by ultra-centrifugation at 15,000 rpm for 2 h at 4 °C using a cooling centrifuge (Beckman, Fullerton, CA, USA). The supernatants were collected and the concentrations of CAPE in the supernatants were determined spectrophotometrically at the predetermined λ_max_ (330 nm).

The entrapment efficiency % (EE%) of CAPE in the liposomes was determined according to the following equation [32]:

$$\mathrm{EE}\%=\frac{\mathrm{TD}-\mathrm{FD}}{\mathrm{TD}}\times 100$$

where EE% is the percentage of CAPE entrapped, FD is the amount of free CAPE in the supernatant and TD is the total amount of CAPE used in the preparation of the NL.

#### 2.3.3. Differential Scanning Calorimetry (DSC)

The thermal analysis of pure materials and the lyophilized optimum CAPE-loaded-NL formulation was determined using Shimadzu differential scanning calorimeter (DSC-50, Kyoto, Japan). Approximately 4 mg samples were sealed in aluminum pans and thermograms were obtained by heating at a constant heating rate of 10 °C/min in the range of 30–300 °C under an inert nitrogen flow (25 mL/min).

#### 2.3.4. Morphological Characterization

The morphology of the optimal CAPE-loaded-NL formulation was determined by transmission electron microscopy (TEM) operated at a voltage of 200 kV. One drop of the nanosuspension was deposited on a copper-gold carbon grid negatively stained with 2% phosphotungstic acid and left to be air-dried at room temperature. Then, the grid was placed in the vacuum chamber of the electron microscope (JEOL-2100, Jeol Ltd., Tokyo, Japan) and images were captured using different magnification powers.

### 2.4. In Vivo Studies

#### 2.4.1. Animals

The in vivo protective effect of the optimal CAPE-loaded-NL formulation was tested using a rat model of acute pancreatitis. The study protocol was approved by the Ethics Committee of Animal Care and Use at the Faculty of Pharmacy, Cairo University, Cairo, Egypt (Approval Number: BC 3087, 27 September 2021). All animal procedures also comply with Directive 2010/63/EU.

The study was conducted on a total of 32 male adult Wistar rats weighing 180–220 g. The animals were brought from the laboratory animal farm of the Egyptian Organization for Biological Products and Vaccines, Cairo, Egypt. The animals were housed in the experimental animal unit of the Faculty of Pharmacy, Cairo University, under controlled conditions of temperature (25 ± 2 °C) and humidity (60 ± 10%) with a 12 h/12 h light/dark cycle. The animals were fed with standard chow diet with free access to water throughout the entire period of the study.

#### 2.4.2. Drug Preparation and Administration

All animals received a single dose/day of free CAPE suspension or CAPE-loaded-NL formulation for seven consecutive days by oral gavage. All suspensions were constituted in a vehicle containing 20% Tween to prevent any particle sedimentation/aggregation and ensure a uniform suspension. All CAPE suspensions were also freshly prepared on the day they were administered, while CAPE-Loaded-NL were lyophilized the day before the first dose and reconstituted in the vehicle containing 20% Tween on the study day right before administration to the rats. Short-term stability studies were carried out for lyophilized powders for at least 10 days at room temperature or 4 °C and results (data not shown) showed non-significant change in the vesicles’ size after redispersion when stored at room temperature or 4 °C. The drug concentrations were adjusted so that each 100 g body weight received 0.5 mL of drug preparation containing the required dose.

#### 2.4.3. Experimental Design

After a one-week acclimatization period, the rats were randomly allocated into four treatment groups of eight rats each (*n* = 8). Group I (Control group) consisted of normal rats which received physiological saline containing 20% Tween 20 by gavage daily for 7 days, in addition to a single intraperitoneal injection of physiological saline 12 h before the seventh oral saline dose. Groups II, III and IV were subjected to acute pancreatitis (AP) induced by a single intraperitoneal injection of 3 g/kg L-ornithine dissolved in physiological saline [11]. Group II (ORN group) received physiological saline containing 20% Tween 20 by oral gavage daily for 7 days, with the L-ornithine injection given 12 h before the seventh oral saline dose. Group III (ORN+CAPES) received free CAPE suspended in saline containing 20% Tween 20 (CAPES) by oral gavage at a dose of 10 μmol/kg/day for 7 days, with the L-ornithine injection given 12 h prior to the seventh CAPES dose. Group IV (ORN+ CAPEL) received the optimal CAPE-loaded-NL (CAPEL) by oral gavage at a dose of 10 μmol/kg/day for 7 days [33], with the L-ornithine injection given 12 h prior to the seventh CAPEL dose.

#### 2.4.4. Blood Sampling and Tissue Preparation

In this case, 24 h following the L-ornithine injection, all rats were sacrificed by decapitation under thiopental anesthesia (50 mg/kg, i.p.) (Sigma-Aldrich Co., St. Louis, MO, USA). Immediately after decapitation, serum was separated for the determination of amylase and lipase activities and insulin level.

The pancreas was excised instantly after decapitation under ice-cold conditions, washed with ice-cold saline, blotted dry and then it was divided into 3 parts. One part was immediately fixed in 10% neutral buffered formalin for 72 h to perform the histological and immunohistochemical examinations. The second and third parts were used to prepare 10% homogenates; the second part was homogenized in 100 mM potassium phosphate buffer (pH 6), containing 1% hexadecyltrimethylammonium bromide, for the determination of MPO activity, and the third part was homogenized in ice-cold saline for other determinations. All samples were stored at −80 °C till use.

#### 2.4.5. Determination of Biochemical Parameters

##### Determination of Serum Amylase and Lipase Activities and Insulin Level

Serum amylase activity was assayed using a colorimetric kit provided by QCA (Amposta, Spain), based on the amylase-dependent liberation of the p-nitrophenol (pNP) colored product, measured colorimetrically at 405 nm, which is proportional to the amount of substrate, ethylidene-G7-pNP 4,6-ethylidene(G7)-p-nitrophenyl(G1)-α,D maltoheptaoside, cleaved by the amylase enzyme. Serum lipase activity was measured using a colorimetric kit obtained from QCA (Amposta, Spain). The principle of the assay is based on the cleavage of the lipase substrate 1,2-O-dilauryl-rac-glycero-3-glutaric acid-(6-methylresorufin)-ester at alkaline pH by the catalytic action of pancreatic lipase to form 1,2-O-dilauryl-rac-glycerol and the unstable compound glutaric acid-(6-methylresorufin)-ester which, in turn, degrades to glutaric acid and methyl-resorufin in that alkaline solution. The color intensity of the red dye formed can be measured at 578 nm and is directly proportional to the lipase activity.

Serum insulin level was determined using a rat insulin enzyme-linked immunosorbent assay (ELISA) kit (Catalog number: ELR-Insulin) provided by RayBiotech Life, Inc. (Norcross, GA, USA), adopting the quantitative sandwich enzyme immunoassay technique, according to the manufacturer’s instructions.

##### Determination of Pancreatic Nuclear Factor Erythroid 2-Related Factor 2 (Nrf2), Heme Oxygenase-1 (HO-1), Tumor Necrosis Factor-α (TNF-α), ADP and ATP Levels

Pancreatic homogenate was used for the assay of nuclear factor erythroid 2-related factor 2 (Nrf2), heme oxygenase-1 (HO-1), tumor necrosis factor-α (TNF-α), ADP and ATP levels by enzyme-linked immunosorbent assay (ELISA) technique.

Nrf2 protein content was assayed in the pancreatic nuclear extract using an ELISA kit (Catalog number: 600590) provided by Cayman Chemical (Ann Arbor, MI, USA). The nuclear fraction was extracted from the pancreatic homogenate by a nuclear extraction kit (Catalog number: 10009277) from Cayman Chemical (Ann Arbor, MI, USA). The method was based on detecting the specific DNA binding activity of Nrf2 by using a specific double stranded DNA sequence containing the Nrf2 response element. Pancreatic HO-1 protein expression was assayed using an ELISA kit (Catalog number: MK124) adopting the quantitative sandwich enzyme immunoassay technique according to the manufacturer’s guidelines (Takara Bio Inc., Shiga, Japan). Pancreatic TNF-α content was determined using an ELISA kit (Catalog number: CSB-E11987r), employing the quantitative sandwich enzyme immunoassay technique according to the manufacturer’s protocol (Cusabio, Wuhan, China). Tissue levels of ATP and ADP were assessed by the competitive enzyme immunoassay technique using ELISA kits (Catalog number: MBS723034 and MBS744390, respectively) obtained from MyBioSource (San Diego, CA, USA), according to the manufacturer’s instructions.

##### Determination of Pancreatic Myeloperoxidase (MPO) Activity

Pancreatic MPO activity was assessed, as a marker of neutrophil infiltration, according to the method of Bradley et al. [34], based on measuring the hydrogen peroxide-dependent oxidation of o-dianisidine, catalyzed by MPO, which results in an increased absorbance at 460 nm.

##### Determination of Pancreatic Oxidative Stress Biomarkers

Tissue lipid peroxidation was assessed by the determination of pancreatic MDA content in the post-nuclear fraction of the 2.3% KCl-treated homogenate by the thiobarbituric acid assay [35]. Tissue glutathione (GSH) level was determined in the protein-free post-nuclear fraction using Ellman’s reagent [5,5′-dithiobis-(2-nitrobenzoic acid)], prepared according to Beutler et al. [36]. The assay is based on the reduction of Ellman’s reagent by the SH-group in GSH and the resulting yellow-colored 5-thio-2-nitrobenzoic acid is measured colorimetrically at 412 nm [36]. Glutathione reductase (GR) activity, which converts oxidized glutathione (GSSG) to the reduced form (GSH), was assayed by measuring the decrease in absorbance at 340 nm due to the oxidation of NADPH [37].

##### Determination of Pancreatic Nitric Oxide (NO_x_) Content

Nitric oxide was determined in the pancreatic tissue homogenate as nitrate (NO_3_) and nitrite (NO_2_), together described as NO_x_, according to the method described by Miranda et al. [38]. Based on the strong association of in vivo nitric oxide output with nitrite/nitrate (NO_x_) levels, the method assesses total nitrite/nitrate via the reduction of any nitrate to nitrite by vanadium followed by the detection of total nitrite, including intrinsic nitrite plus that resulting from the reduction of nitrate, by Griess reagent, prepared as described in the method. Griess reaction involves the formation of an azo derivative through the diazotization of sulfanilamide by acidic nitrite followed by coupling with the bicyclic amine, N-(1-naphthyl) ethylenediamine. The formed chromophore is measured colorimetrically at 540 nm.

##### Determination of Protein Content in Pancreatic Tissue

The protein content of pancreatic tissue was determined according to the method of Lowry et al. [39], using the Folin phenol reagent, prepared as described in the method. The color developed from the reduction of phosphomolybdic-phosphotungstic reagent in Folin reagent by copper-treated protein in alkaline medium is measured colorimetrically at 500 nm.

##### Histological and Immunohistochemical Examination

Pancreatic tissue samples were flushed and fixed in 10% neutral buffered formalin for 72 h. Samples were trimmed and processed in serial grades of alcohol, cleared in xylene, infiltrated and embedded into Paraplast Plus tissue embedding media (Leica Biosystems, Richmond, IL, USA). 5-μm thick tissue sections were cut by a rotary microtome (Leica RM2235, Leica Biosystems, Nussloch, Germany) and mounted on glass slides. Tissue sections were stained by hematoxylin and eosin and were then examined under the light microscope. All standard procedures for sample fixation and staining were performed as previously described [40].

The histological grading of pancreatic damage was performed, as previously described [41], using a scale ranging from 0 to 3 as follows: 0 = absent, 1 = records in less than 15% of examined tissue sections (mild), 2 = records in 15–35% of examined tissue sections (moderate), and 3 = records in more than 35% of examined tissue sections (severe).

The pancreatic expression of NFκB-p65, cleaved caspase-3, Bcl-2 and Bax was evaluated by immunohistochemical staining of 5-μm thick paraffin-embedded tissue sections as previously described [42,43]. First, tissue sections were processed for antigen retrieval and peroxidase quenching by treatment with 0.03% trypsin at 37 °C for 1 h followed by treatment with 0.3% H_2_O_2_ for 20 min. The tissue sections were then incubated with anti-NF-κB p65 (Catalog number: RB-1638-P0, Thermo Fisher Scientific, Carlsbad, CA, USA), diluted 1:100, anti-cleaved caspase-3 (Catalog number: Asp175, Cell Signaling Technology Inc., Beverly, MA, USA), diluted 1:200, anti-Bcl2 (Catalog number: PA5-27094, Thermo Fisher Scientific, Carlsbad, CA, USA), diluted 1:100, and anti-Bax (Catalog number: 33-6600, Thermo Fisher Scientific, Carlsbad, CA, USA), diluted 1:100. After washing with PBS, the tissue sections were incubated with a secondary antibody-horseradish peroxidase (HRP) Envision kit (Dako, Copenhagen, Denmark) for 20 min, washed and incubated with 3,3′-diaminobenzidine (Dako, Copenhagen, Denmark) for 15 min. After another washing with PBS, the tissue sections were counterstained with hematoxylin, dehydrated and cleared in xylene then cover-slipped for microscopic examination. 6 non-overlapping fields were randomly selected and scanned for the determination of the area percentage of immunoexpression levels of NF-κB p65, cleaved caspase-3, Bcl-2 and Bax. Image visualization and analysis were performed using the Leica application module for histological analysis attached to a full HD microscopic imaging system (Leica Microsystems GmbH, Wetzlar, Germany).

### 2.5. Statistical Analysis

The normality of data was tested by Shapiro−Wilk normality test. Data are given as the mean ± standard deviation (SD), and the statistical comparisons were conducted using one-way analysis of variance (ANOVA), followed by Tukey’s multiple comparisons test. Statistical analyses were performed using the GraphPad Prism software, version 7.04 (San Diego, CA, USA). Differences among the experimental groups were considered statistically significant at *p* < 0.05.

## 3. Results and Discussion

### 3.1. Preparation and Optimization of CAPE-Loaded-NL

Liposomes (LP) are vesicles in the nanometer size range formed by self-assembly of phospholipids molecules in solution. The liposomal membrane is mainly composed of a lipid bilayer enclosing an internal aqueous core [44]. LP represent a significant vesicular carrier system for therapeutic effectiveness in terms of duration of action, decrease in dosing frequency, and delivering encapsulated actives at a higher efficacy and lower toxicity [26].

In this study, CAPE-loaded-NL were optimized for vesicle size (VS) and entrapment efficiency (EE%) using three different edge activators with the target set at the smallest VS and an EE% of no less than 75%. The size of the vesicles plays a crucial role in the interaction between the vesicles and biological barriers [45]. It is acknowledged that the smaller the VS is, the greater the extent of uptake will be. Two biological events are usually referred to the uptake through a biological membrane, e.g., intestinal epithelium, through which the vesicles move from the site of administration to the bloodstream, and cellular internalization through cell membrane on single cell level [46].

The VS of the prepared CAPE-loaded-NL ranged from 309–1211 nm and the EE% ranged from 77% to 93% indicating the significant impact of the edge activator (EA) on these formulation variables. Table 1 shows the effect of the type of EA on the VS, PDI, EE%, and ZP of the prepared CAPE-loaded-NL. Statistical analysis revealed that the type of EA had significant effects on VS, PDI, EE% and ZP (*p* < 0.05 for all). CAPE-loaded-NL prepared using sodium cholate (SC) as an EA showed larger VS (1211.7 ± 433.2 nm) and higher EE% compared to those prepared employing Tween 80 and sodium deoxycholate (SDC) indicating that the factor that has positively contributed to increasing the EE% has as well significantly increased the VS. These findings are consistent with published research in which it was reported there is a direct relationship that correlates the size of the vesicles with the amount of drug entrapped (EE%) as the distance between the bilayers increases due to drug inclusion in the hydrophobic area within the vesicles [47]. Similar results were also obtained by Basha et al. in a study on the design of surfactant-based nanovesicles for ocular delivery of clotrimazole [48]. Statistical analysis revealed that the larger vesicles obtained using sodium cholate as EA were associated with significantly wider size distribution (*p* < 0.05), while smaller vesicles prepared using Tween 80 or SDC were associated with lower PDI values. Successful formulation of stable and efficient CAPE-loaded-NL requires the preparation of homogenous (monodisperse) populations of liposomes of a certain size.

ZP is a measure of the overall charges acquired by the vesicles and is correlated with the in vitro and in vivo stability of vesicular formulations. A minimum zeta potential of ±30 mV is required to obtain physically stable liposomal suspensions stabilized by electrostatic repulsion only [49]. The ZP values of all CAPE-loaded-NL were negative, ranging from −47 mV to −57 mV. The high ZP acquired by all NL formulations contribute to the effective stabilization of the nanosuspensions due to electrostatic repulsion thus preventing vesicles aggregation.

According to the statistical analysis, SDC was the most successful EA in producing the smallest CAPE-loaded-NL (309 nm) with acceptable EE% (87%), acceptable PDI value (0.46) and high ZP (−47 mV). This optimal formulation may provide a promising nanocarrier for CAPE and therefore was selected for further investigations. The optimal formulation was coded CAPEL.

### 3.2. Differential Scanning Calorimetry (DSC)

**Figure 1:**
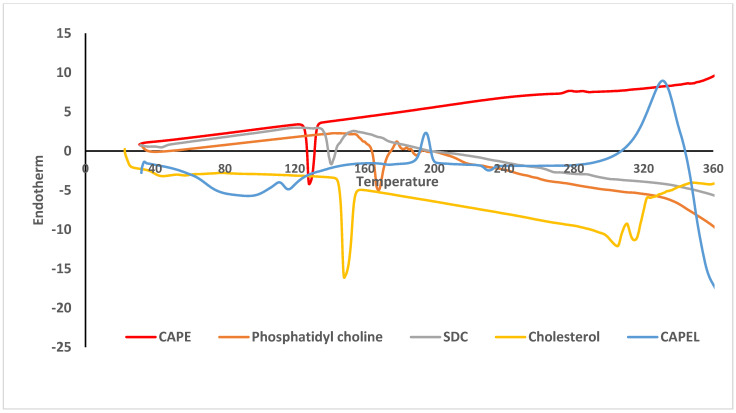
Differential scan calorimetry (DSC) thermogram of caffeic acid phenethyl ester (CAPE)-optimal liposomal formulation (CAPEL) in comparison to free caffeic acid phenethyl ester (CAPE), phosphatidyl choline, sodium deoxycholate (SDC) and cholesterol (*n* = 1).

DSC is a tool used to investigate the crystalline or amorphous nature of the drug within the developed formulation and to elucidate any possible interactions with other ingredients. Figure 1 shows the DSC thermograms of free CAPE, phosphatidyl choline, cholesterol, sodium deoxycholate (SDC) and lyophilized CAPEL. The DSC thermograms of pure CAPE showed a strong endothermic peak at nearly 128 °C corresponding to its melting transition point. The DSC thermogram of phosphatidyl choline revealed the presence of a broad endotherm starting at 167 °C probably due to loss of water molecules followed by an exothermic recrystallization peak at 366 °C. The DSC thermogram of cholesterol showed a sharp endothermic peak at 148 °C while that of SDC revealed an endothermic peak at 365 °C. For CAPEL, the characteristic peak of CAPE completely disappeared which probably signifies that CAPE was either entrapped inside the liposomes [50] or completely transformed from crystalline form to molecular state in the surfactant mixture. Nasr et al. reported that the absence of the crystalline melting peak of the drug after encapsulation might also indicate the presence of a strong interaction between the surfactant bilayers of the vesicles and the entrapped drug [51].

### 3.3. Morphological Characterization

**Figure 2:**
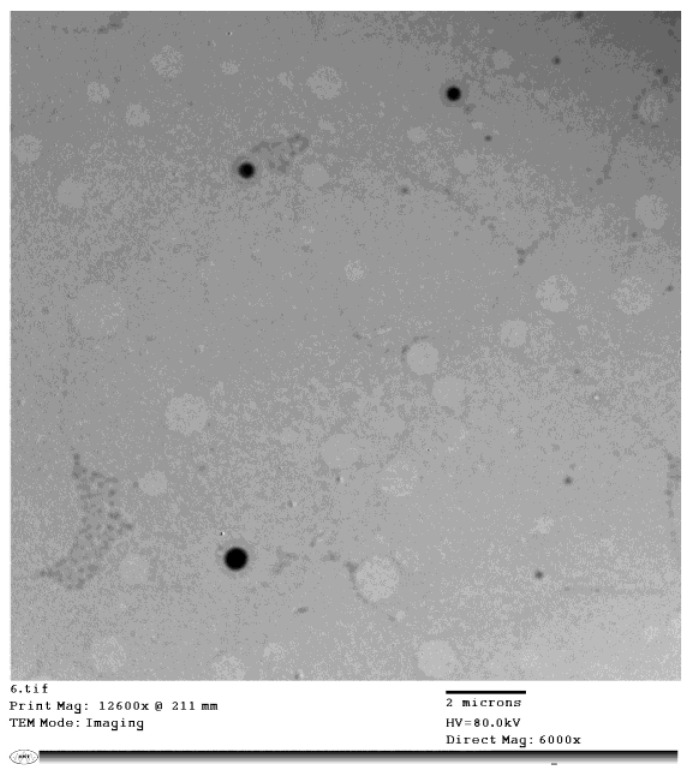
Morphology and size of caffeic acid phenethyl ester (CAPE)-optimal liposomal formulation (CAPEL) using transmission electron microscope (TEM) (*n* = 1).

The TEM micrographs of CAPEL showed unilamellar vesicles with spherical shape. The vesicles were characterized by smooth surface with narrow size distribution (Figure 2). The figure clearly shows that the size of the vesicles observed in the micrographs is in accordance with the data obtained by particle size analysis. The slightly smaller VS obtained by TEM compared to dynamic light scattering technique (DLS) could be due to the reported dehydration effect of TEM imaging [52].

### 3.4. In Vivo Studies

Despite the wide spectrum of beneficial biological effects of CAPE and its promising ameliorating potential in several models of AP, its efficacy is constrained by its poor water solubility following oral administration and its instability in the plasma. To circumvent the bioavailability challenge associated with the use of CAPE, we herein evaluate the efficacy of the developed optimal CAPE-loaded-NL formulation (CAPEL) in an ornithine-induced rat model of AP in comparison to free CAPE suspension (CAPES). The mechanism underlying L-ornithine-induced pancreatitis is not fully elucidated. It probably involves the dysregulation of pancreatic polyamine levels resulting from the large dose of ornithine [11]. L-ornithine is the substrate of ornithine decarboxylase, the initial and rate-limiting enzyme in the biosynthesis of polyamines, which are crucial for normal cell development. A dysregulation in pancreatic polyamine levels, in response to a large dose of L-ornithine or L-arginine (a precursor of L-ornithine), is likely to cause a suppression of DNA and protein synthesis, eventually leading to the death of pancreatic acini [11].

#### 3.4.1. Effect of Pretreatment with CAPEL on Biochemical Parameters

##### Effect on Serum Amylase and Lipase Activities and Insulin Level

**Figure 3:**
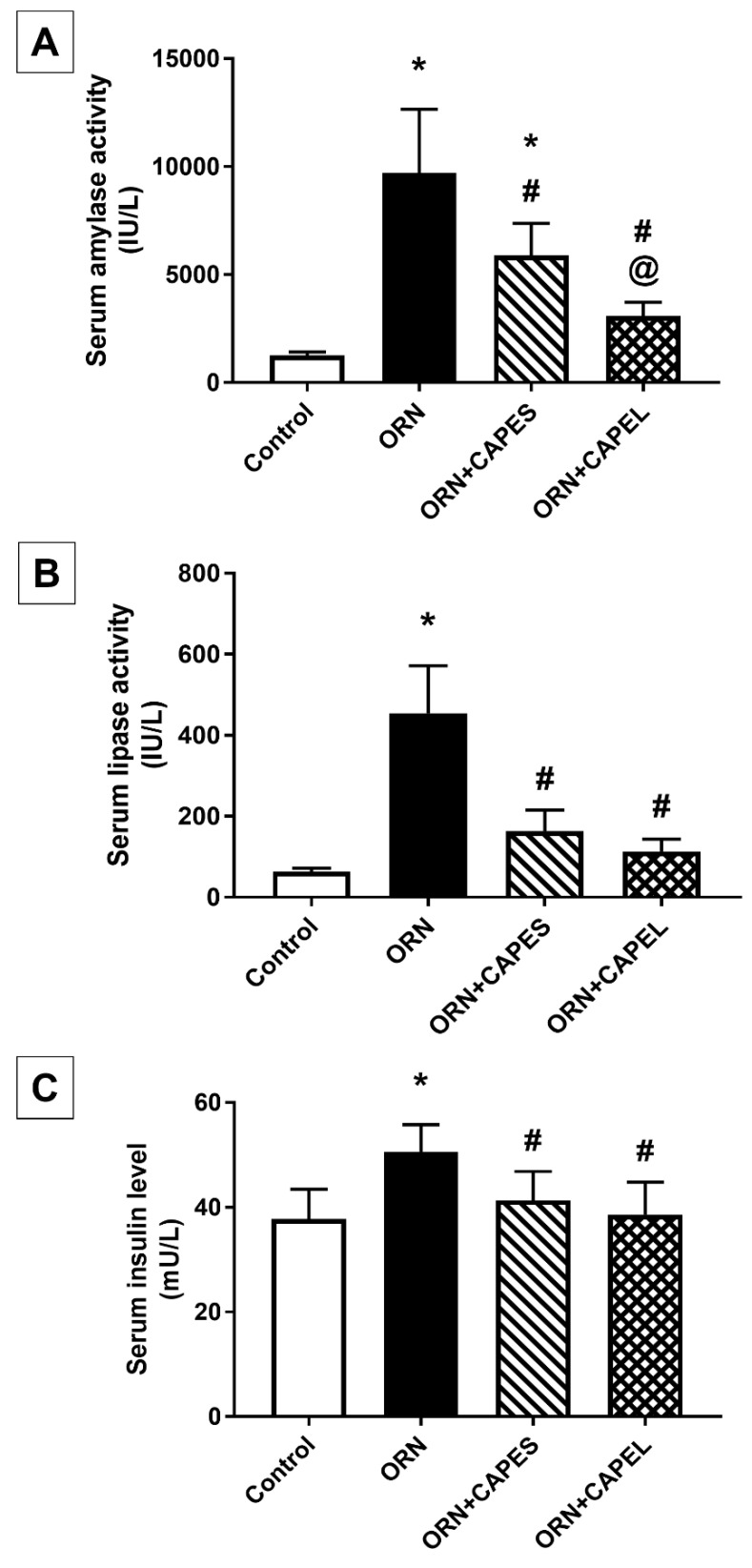
Effect of administering caffeic acid phenethyl ester (CAPE)-optimal liposomal formulation (CAPEL) on serum amylase and lipase activities and insulin level in rats with ornithine-induced acute pancreatitis. (**A**) Serum amylase activity. (**B**) Serum lipase activity. (**C**) Serum insulin level. Data are expressed as mean ± S.D. (*n* = 8). *, # and @ indicate significant difference at *p* < 0.05 from control, ORN and ORN+CAPES groups, respectively (one-way ANOVA followed by Tukey’s multiple comparisons test). ORN, ornithine; CAPES, free caffeic acid phenethyl ester (CAPE) suspension; CAPEL, caffeic acid phenethyl ester (CAPE)-optimal liposomal formulation.

The elevated serum activities of the pancreatic digestive enzymes amylase and lipase in the ornithine-treated group are in harmony with previous reports from various models of AP [6,11], and indicate pancreatic tissue damage. Serum amylase and lipase activities, and insulin level in the four study groups are shown in Figure 3. Serum amylase and lipase activities were significantly increased in the ORN group showing 6.7 and 6.2-fold elevations, respectively, when compared with the control group. Administration of free CAPE suspension (CAPES) and the optimal CAPE-loaded-NL formulation (CAPEL) to ornithine-treated rats resulted in significant 39.4% and 68.3% reduction in serum amylase activity and 64% and 75% reduction in lipase activity, respectively, relative to the ORN group. Importantly, the CAPEL group showed significantly greater improvement than the CAPES group, as manifested by 47.6% lower amylase activity compared to CAPES-treated rats, thereby restoring its activity back to normal, a precedence that implies its enhanced efficacy. Lipase activity, on the other hand, was normalized by both treatments with an apparently more prominent effect exerted by CAPEL, resulting in a 30.8% lower value than that attained by CAPES treatment (Figure 3A,B).

The beneficial effect of CAPE in AP and pancreatic injury has been previously demonstrated in vancomycin, cerulein and cisplatin-induced models [15,33,53] showing significant lowering of serum amylase and lipase activities, while no direct effect was reported for CAPE on serum amylase or lipase activities in normal rats [20,53]. However, no studies so far have addressed the possible benefit of incorporating CAPE into a nano-based formulation to enhance its oral bioavailability and hence its efficacy in any model of AP.

The results also showed that ornithine induced a significant 33.9% elevation in serum insulin level as compared to the control value. The elevated serum insulin level associated with pancreatitis is in accordance with previously reported research [54] and could be attributed to the destruction of pancreatic islets caused by pancreatitis leading to β-cell injury and excessive release of stored insulin. Such pancreatitis-related destruction of insulin-producing cells was markedly relieved by the administration of CAPE, indicating mitigated damage of the islets and conservation of the remaining pancreatic islet β-cell function thereby restricting the excessive release of stored insulin. No significant difference was observed between the CAPES and CAPEL treated groups in serum insulin level. Both CAPES and CAPEL treatments succeeded in normalizing the increased insulin level by achieving significant 18.2% and 23.7% decrements, respectively, compared to the insulin level of the ORN group (Figure 3C).

##### Effect on Pancreatic Oxidative Stress Biomarkers and Nrf2 Signaling

**Figure 4:**
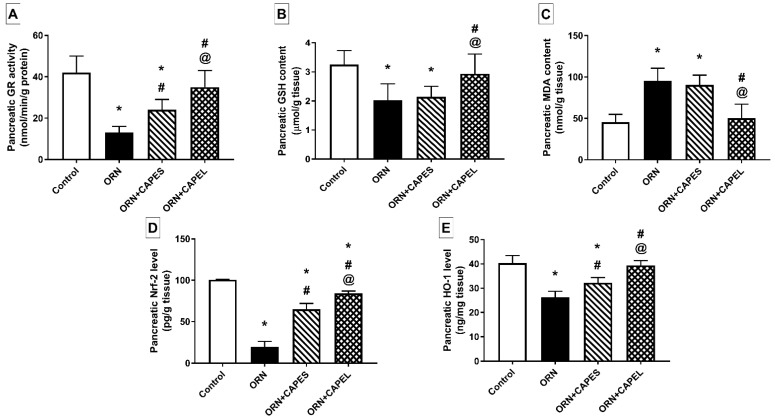
Effect of administering caffeic acid phenethyl ester (CAPE)-optimal liposomal formulation (CAPEL) on pancreatic oxidative status and Nrf2 signaling in rats with ornithine-induced acute pancreatitis. (**A**) Pancreatic glutathione reductase (GR) activity. (**B**) Pancreatic glutathione (GSH) content. (**C**) Pancreatic malondialdehyde (MDA) content. (**D**) Pancreatic nuclear factor erythroid 2-related factor 2 (Nrf2) level. (**E**) Pancreatic heme oxygenase-1 (HO-1) level. Data are expressed as mean ± S.D. (*n* = 8). *, # and @ indicate significant difference at *p* < 0.05 from control, ORN and ORN+CAPES groups, respectively (one-way ANOVA followed by Tukey’s multiple comparisons test). ORN, ornithine; CAPES, free caffeic acid phenethyl ester (CAPE) suspension; CAPEL, caffeic acid phenethyl ester (CAPE)-optimal liposomal formulation.

Oxidative stress has been demonstrated to play a cardinal role in the pathogenesis of AP [12]. In accordance with previous reports [6,53], rats with AP showed significantly diminished activity of glutathione reductase (GR), significantly decreased levels of glutathione (GSH), the endogenous antioxidant, and significantly elevated levels of malondialdehyde (MDA), the end product of lipid peroxidation. Induction of pancreatitis elicited a marked 69% and 37.5% decline in pancreatic GR activity and GSH content, respectively, and a notable 110% increase in the pancreatic level of MDA compared to the corresponding control values. CAPEL-treated rats showed markedly alleviated pancreatic oxidative stress as manifested by significant 1.7-fold increase in GR activity, 44.3% elevation in GSH level and 47.1% decrease in MDA content compared to the corresponding values in the ORN group. These findings indicate attenuated lipid peroxidation and enhanced endogenous antioxidant defense. On the other hand, CAPES administration failed to significantly alter the ornithine-induced GSH and MDA aberrations, despite significantly raising the GR activity by 85% compared to the ORN group (Figure 4A–C). The observed antioxidant action of CAPE could be explained based on its stimulatory effect on Nrf2/HO-1 signaling as described below.

Nuclear factor erythroid 2-related factor 2 (Nrf2), a member of the NF-E2 family of nuclear basic leucine zipper transcription factors, regulates the transcription of an array of ROS-detoxifying enzymes that act to protect against oxidative stressors [17]. Decreased pancreatic Nrf2 expression has been reported in several models of AP [6,7]. The decreased protein expression of Nrf2 observed in the ORN group could be attributed to the down-regulation of Nrf2 gene expression as previously reported in a cerulein-induced AP model [7]. In addition, oxidative stress associated with AP has been reported to deplete Nrf2 [6]. The down-regulation of pancreatic Nrf2, observed in the ORN group, justifies the corresponding lowered expression of its downstream effector, HO-1.

Pancreatic Nrf2 level was markedly depleted in the ornithine-treated rats as manifested by a significant 80.5% reduction in its level compared to the control value. Such effect was significantly counteracted by CAPE administration which resulted in 3.3- and 4.3-fold values in CAPES and CAPEL-treated rats, respectively, compared to that of the ORN group. Importantly, CAPEL was superior to CAPES in replenishing the pancreatic Nrf2 level as evidenced by a 29.2% significantly higher Nrf2 level in the CAPEL-treated group compared to the CAPES-treated group (Figure 4D). The effect of CAPE on Nrf2 expression is in agreement with a previous study in which CAPE was shown to promote the synthesis and activation of Nrf2 in hepatic stellate cells-T6 (HSC-T6), cultured in vitro, via increasing Nrf2 gene expression and promoting Nrf2 protein translocation from the cytosol to the nucleus, respectively [19]. The downstream effector, HO-1, was significantly down-regulated in response to ornithine administration showing an expression that was 35% lower than the control value. Administration of CAPES and CAPEL significantly increased pancreatic HO-1 protein levels by 23.1% and 50.4%, respectively, compared to the ORN group, but only CAPEL treatment was able to normalize the HO-1 level, achieving a 22.2% significantly higher value than that of the CAPES-treated group (Figure 4E). The induction of HO-1 by polyphenolic antioxidants, including CAPE, is presumably mediated by the activation of the Nrf2/ARE pathway via interacting with the thiol groups in keap1, thereby liberating Nrf2 from the Nrf2-keap1 complex. The released Nrf2 is consequently translocated to the nucleus, binds to the HO-1 ARE and enhances its transcription [55]. The proposed implication of CAPE-induced HO-1 in counteracting oxidative stress is in accordance with a former report linking the antioxidant effect of CAPE to its potent ability to up-regulate HO-1 [56].

##### Effect on Pancreatic NF-κB Signaling and Inflammation

The substantially elevated levels of NF-κB p65 and TNF-α depict an intense state of inflammation in the pancreatic tissue of ornithine-treated rats. The up-regulation of pancreatic NF-κB p65 expression indicates NF-κB activation, and could be attributed to the degradation of pancreatic IκB proteins as formerly reported in an L-ornithine-induced model of AP [11]. In accord with former studies [6,7], pancreatic TNF-α level significantly increased in rats with AP corresponding to the observed NF-κB activation.

**Figure 5:**
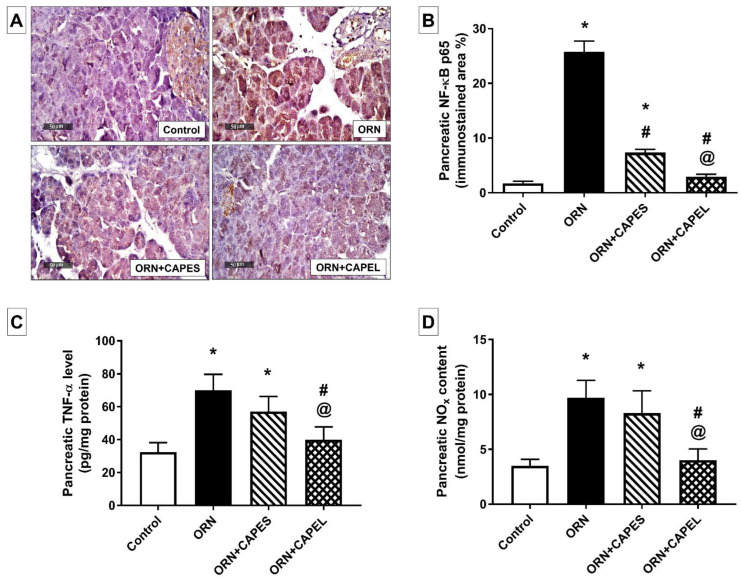
Effect of administering caffeic acid phenethyl ester (CAPE)-optimal liposomal formulation (CAPEL) on pancreatic NF-κB signaling and inflammatory status in rats with ornithine-induced acute pancreatitis. (**A**) Representative photomicrographs of the immunohistochemical evaluation of pancreatic nuclear factor kappa B (NF-κB) p65 protein expression in the different study groups (×400 magnification). (**B**) Quantification of NF-κB p65 immunostaining in pancreatic tissue. Values are the mean ± S.D. of the area percentage of NF-κB p65-immunostaining to the total area of the microscopic field across six non-overlapping fields/section (*n* = 8). (**C**) Pancreatic tumor necrosis factor-alpha (TNF-α) level. (**D**) Pancreatic nitrite/nitrate (NO_x_) level. Data are expressed as mean ± S.D. (*n* = 8). *, # and @ indicate significant difference at *p* < 0.05 from control, ORN and ORN+CAPES groups, respectively (one-way ANOVA followed by Tukey’s multiple comparisons test). ORN, ornithine; CAPES, free caffeic acid phenethyl ester (CAPE) suspension; CAPEL, caffeic acid phenethyl ester (CAPE)-optimal liposomal formulation.

Rats in the ORN group exhibited a dramatic 14-fold elevation in pancreatic NF-κB p65 expression compared to the control group. CAPEL administration resulted in considerable 88.5% reduction in pancreatic NF-κB p65 expression compared to its expression in the ORN group. The CAPEL treatment effect was also significantly more potent compared to CAPES treatment which resulted in only 71.5% decrement (Figure 5A,B).

The pro-inflammatory cytokine, TNF-α, was significantly elevated in the pancreatic tissue of rats with pancreatitis (ORN group) reaching 2.15-fold the control value. Such inflammatory status was significantly mitigated by CAPEL administration which resulted in 42.9% decline in pancreatic TNF-α level compared to the ORN group. On the other hand, CAPES administration resulted in an apparent non-significant 18.6% decrement in pancreatic TNF-α level compared to the ORN group value (Figure 5C).

Rats in the ORN group exhibited a significant 1.8-fold increase in pancreatic nitrite/nitrate (NO_x_) content compared to the control group. The concurrent elevation of NF-κB p65 and NO_X_ levels suggests an active NF-κB-iNOS-NO signaling axis. As a transcriptional activator of various inflammatory genes including iNOS, NF-κB activation observed in the ORN group may underlie the corresponding elevation in total nitrite/nitrate level via the induction of iNOS overexpression. Treatment with CAPEL resulted in the normalization of the NO_x_ content through a significant 58.8% reduction in the NO_x_ level when compared with the ORN group. The effect of CAPEL treatment on pancreatic NO_x_ level was significantly higher compared to CAPES treatment which failed to achieve a significant improvement in this regard (Figure 5D).

It is worth mentioning that besides the reported ability of CAPE to inhibit NF-κB DNA binding activity, CAPE treatment has also been shown to decrease the protein expression of NF-κB1 (p50) and RelA (p65) both in vitro and in vivo. These data reinforce the notion that CAPE may act at both transcriptional and post-transcriptional levels to attenuate NF-κB signaling via suppressing its ability to regulate gene transcription or down-regulating its protein expression [16].

In the context of the anti-inflammatory effects of CAPE, its anti-thrombotic capability may also be a possible contributor. There is a close relationship between clotting and the development of inflammation. Coagulation stimulates the development of inflammation, and at the same time, inflammation activates the coagulation cascade [57]. Previous studies have shown that anti-coagulative factors such as heparin [58], warfarin [59] and acenocoumarol [60] exhibit protective and therapeutic effects in AP. In light of previous research showing that CAPE inhibits endothelial tissue factor (TF) protein expression and activity at the posttranscriptional level [61], this effect suggests an anti-thrombotic potential of CAPE in AP.

The role of pancreatic blood flow in the development and the course of AP should not be ruled out. Pancreatic ischemia may be a primary cause of AP, and also plays an essential role in the development of severe forms of the disease [62]. While reduction in pancreatic blood flow leads to aggravation of the severity of AP, improvement of pancreatic blood flow reduces the disease severity and accelerates pancreatic recovery [59,63,64,65]. Although the effect of CAPE on pancreatic blood flow in AP is unknown, it has been shown to alleviate mesenteric ischemia/reperfusion injury [18], suggesting that this mechanism may contribute to the protective effect of CAPE on the pancreas in this condition.

##### Effect on Pancreatic Apoptosis

Apoptosis driven by oxidative stress has been demonstrated in the pancreas of patients with AP [66]. CAPE was previously reported to exert an anti-apoptotic effect by blocking ROS formation and by inhibiting caspase activity [67]. CAPE was also shown to revert the down-regulation of Bcl-2 and the up-regulation of Bax in a cellular model of Parkinson’s disease [68].

**Figure 6:**
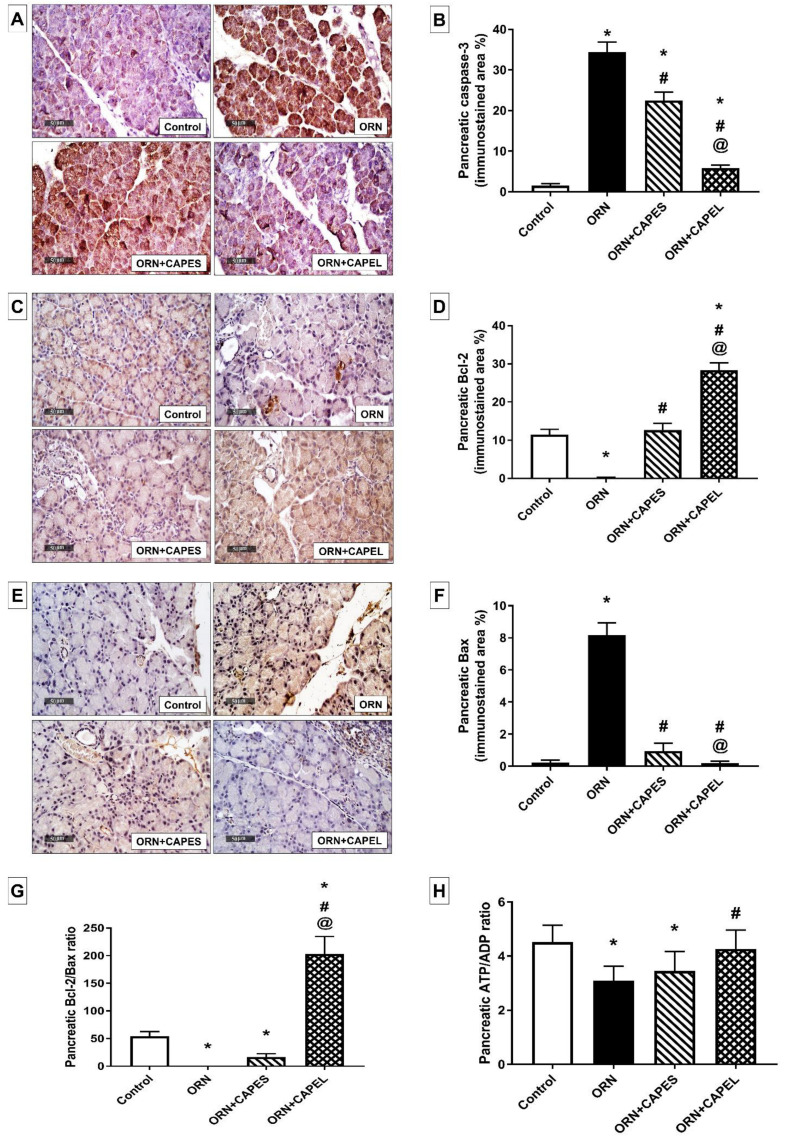
Effect of administering caffeic acid phenethyl ester (CAPE)-optimal liposomal formulation (CAPEL) on pancreatic apoptosis and energy status in rats with ornithine-induced acute pancreatitis. (**A**) Representative photomicrographs of the immunohistochemical evaluation of pancreatic cleaved caspase-3 protein expression in the different study groups (×400 magnification). (**B**) Quantification of cleaved caspase-3 immunostaining in pancreatic tissue. (**C**) Representative photomicrographs of the immunohistochemical evaluation of pancreatic Bcl-2 protein expression in the different study groups (×400 magnification). (**D**) Quantification of Bcl-2 immunostaining in pancreatic tissue. (**E**) Representative photomicrographs of the immunohistochemical evaluation of pancreatic Bax protein expression in the different study groups (×400 magnification). (**F**) Quantification of Bax immunostaining in pancreatic tissue. Values in (**B**,**D**,**F**) are the mean ± S.D. of the area percentage of cleaved caspase-3, Bcl-2 and Bax immunostaining, respectively, to the total area of the microscopic field across six non-overlapping fields/section (*n* = 8). (**G**) Pancreatic Bcl-2/Bax ratio. (**H**) Pancreatic ATP/ADP ratio. Data are expressed as mean ± S.D. (*n* = 8). *, # and @ indicate significant difference at *p* < 0.05 from control, ORN and ORN+CAPES groups, respectively (one-way ANOVA followed by Tukey’s multiple comparisons test). ORN, ornithine; CAPES, free caffeic acid phenethyl ester (CAPE) suspension; CAPEL, caffeic acid phenethyl ester (CAPE)-optimal liposomal formulation.

As depicted in Figure 6A–G, rats in the ORN group displayed substantial enhancement of pancreatic apoptosis as evidenced by the immunohistochemically detected significant overexpression of cleaved caspase-3, the key executioner of apoptosis, in their pancreatic tissue. Immunohistochemical analysis of pancreatic tissue from the ORN group rats also revealed significant overexpression of the pro-apoptotic Bax and significant reduction in the expression of the anti-apoptotic Bcl-2. The mechanism underlying the enhanced apoptosis observed in the ORN group probably involves the disruption of pancreatic polyamine levels due to the large dose of L-ornithine [11]; both elevated and reduced levels of polyamines have been involved in mediating apoptosis [69]. ROS generated during the course of pancreatitis are important mediators of apoptosis [70] and mitochondria, as an important source of ROS in pancreatic acinar cells, play a vital role in the process of apoptosis. Mitochondrial dysfunction evoked by oxidative stress may result in the release of cytochrome c and other apoptogenic mediators from the injured mitochondria and, thereafter, caspase activation, eventually executing apoptotic cell death [70]. This apoptotic surge was significantly mitigated by CAPE administration, in both forms, resulting in 34.7% and 83% lower caspase-3 expression in the CAPES-treated and CAPEL-treated groups, respectively, compared to the ORN group. Here again, the CAPEL treatment was significantly more potent than CAPES treatment as evidenced by a 74% lower caspase-3 immunoexpression than that observed in the CAPES group. Furthermore, substantial suppression of Bax expression and potentiation of Bcl-2 expression were achieved by both CAPE treatments with more pronounced effects being exerted by CAPEL as manifested by 80.7% lower Bax and 123% higher Bcl-2 expression compared to the corresponding values in the CAPES group. Such effects considerably boosted the Bcl-2/Bax ratio in CAPEL-treated rats compared to both the ORN and the CAPES-treated groups. The augmented anti-apoptotic effect of CAPEL, compared to CAPES, could be attributed to its superior ability to reverse pancreatitis-associated oxidative stress.

The energy status of the different groups was evaluated by the determination of the pancreatic energy markers, ATP and ADP. Metabolic alterations occur during AP. A variety of proinflammatory cytokines, presumably triggered via NF-κB activation as observed in the current study, raise the basal metabolic rate, thereby increasing energy consumption [71]. An increase in inorganic phosphate with a concomitant decrease in ATP levels has been reported in alcoholic AP and taurocholate AP in rats [72]. In addition to increased energy consumption during the course of AP, the compromised energy status could also be associated with disrupted mitochondrial function and subsequently impaired mitochondrial ATP production.

As illustrated in Figure 6H, rats in the ORN group showed a significant 31.6% reduction in pancreatic ATP/ADP ratio, an effect which was effectively reversed by CAPEL administration which resulted in a 38.2% significantly higher ratio compared to the ORN group thus indicating an improved energy status. On the other hand, CAPES administration failed to exert any significant effect on the ATP/ADP ratio. In context, a previous study reported that pre-treatment with caffeic acid derivatives before *tert*-butyl hydroperoxide (*t*-BHP) exposure maintained the mitochondrial oxygen consumption rate and ATP content in injured HepG2 cells [17].

#### 3.4.2. Effect of Pretreatment with CAPEL on Pancreatic Histological Aberrations and Neutrophil Infiltration

The results of the present study revealed that the induction of AP triggered substantial neutrophil infiltration in the pancreas as verified by its significantly elevated myeloperoxidase (MPO) activity. This observation is consistent with the inflammatory cell infiltration portrayed in the histological examination. The histologically observed neutrophil infiltration in pancreatic tissue of rats in the ORN group is in accordance with previously reported infiltration of neutrophil granulocytes and monocytes 18 to 36 h after 3 g/kg L-ornithine injection [11]. Elevated pancreatic MPO activity was previously reported in several rat models of AP [11,20,53]. The enhanced neutrophil infiltration could be attributed to the intense inflammatory upsurge associated with pancreatitis as revealed by the observed NF-κB activation which, in turn, plays an important role in the activation of inflammatory mediators (ICAM-1, TNF-α) [8].

**Figure 7:**
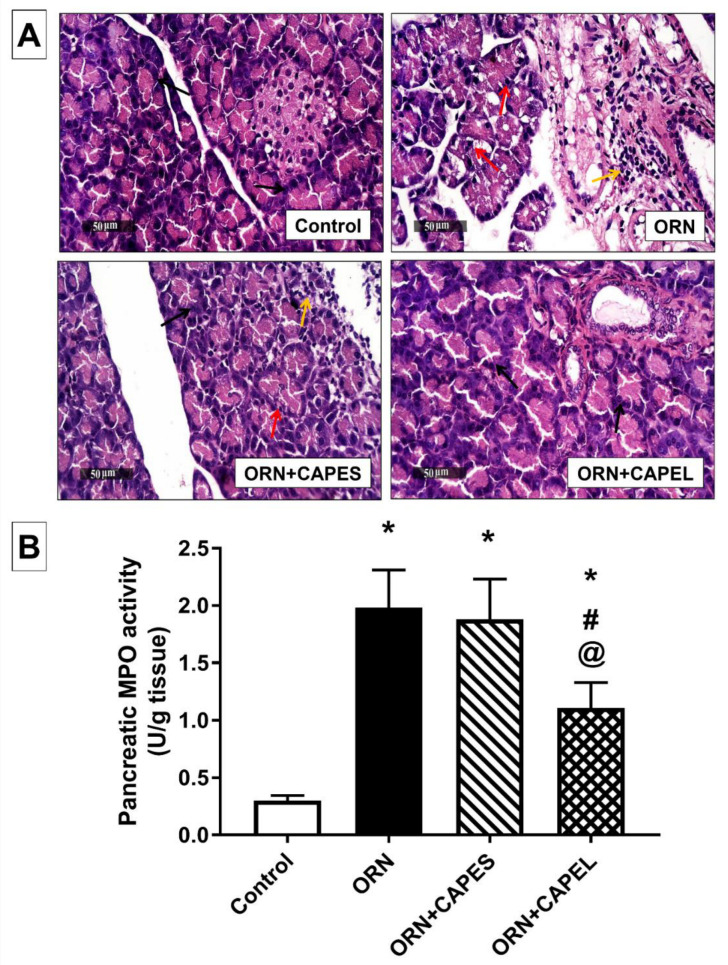
Effect of administering caffeic acid phenethyl ester (CAPE)-optimal liposomal formulation (CAPEL) on pancreatic histological changes and neutrophil infiltration in rats with ornithine-induced acute pancreatitis. (**A**) Representative photomicrographs of the histological examination of pancreatic tissue from the different study groups using H&E stain (×400 magnification): Control samples showed normal histological architecture of pancreatic parenchyma with intact well-organized pancreatic acini in different lobules (arrow), delicate interlobular connective tissue septa and normal intercellular tissue with intact vasculature. ORN samples showed many degenerated lining secretory cells of pancreatic acini with many pyknotic nuclei (red arrow) accompanied with moderate mononuclear interstitial as well as perivascular inflammatory cell infiltration (yellow arrow). ORN+CAPES samples showed higher records of apparently intact pancreatic acini with intact cellular details (black arrow) and fewer focal records of degenerative changes (red arrow) compared to the ORN samples. However, persistence of moderate interstitial inflammatory cell infiltration was observed (yellow arrow). ORN+CAPEL samples showed more apparently intact well-organized pancreatic parenchyma with apparently intact glandular epithelium (black arrow) as well as interstitial tissue with minimal records of inflammatory cell infiltration. (**B**) Pancreatic myeloperoxidase (MPO) activity. Data are expressed as mean ± S.D. (*n* = 8). *, # and @ indicate significant difference at *p* < 0.05 from control, ORN and ORN+CAPES groups, respectively (one-way ANOVA followed by Tukey’s multiple comparisons test). ORN, ornithine; CAPES, free caffeic acid phenethyl ester (CAPE) suspension; CAPEL, caffeic acid phenethyl ester (CAPE)-optimal liposomal formulation.

Figure 7A depicts the histological observations detected in the pancreatic tissues of the different treatment groups. Microscopic examination of pancreatic tissue samples from the control group revealed normal histological features of pancreatic parenchyma with intact well-organized pancreatic acini in different lobules with delicate interlobular connective tissue septa and normal intercellular tissue with intact vasculature. Pancreatic tissue samples from the ORN group showed many degenerated lining secretory cells of pancreatic acini with many pyknotic nuclei accompanied with moderate mononuclear interstitial as well as perivascular inflammatory cell infiltrates. Pancreatic tissue samples from CAPES group showed higher records of apparently intact pancreatic acini with intact cellular details and few focal records of degenerative changes when compared with the ORN group. However, persistence of moderate interstitial inflammatory cell infiltrates was observed. Pancreatic tissue samples from CAPEL group displayed more apparently intact well-organized pancreatic parenchyma with apparently intact glandular epithelium as well as interstitial tissue with minimal records of inflammatory cell infiltrates. The histological grading of pancreatic damage in the different study groups is summarized in Table 2.

Values represent the predominant histological grading in each experimental group (*n* = 8). ORN, ornithine; CAPES, free caffeic acid phenethyl ester (CAPE) suspension; CAPEL, caffeic acid phenethyl ester (CAPE)-optimal liposomal formulation. 0 = absent, 1 = records in less than 15% of examined tissue sections (mild), 2 = records in 15-35% of examined tissue sections (moderate), and 3 = records in more than 35% of examined tissue sections (severe).

In agreement with the histologically observed inflammatory cell infiltration, considerable neutrophil infiltration was detected in the pancreatic tissue of the ORN group as evidenced by a significant 5.6-fold elevation in pancreatic MPO activity compared to the control group. In accordance with the histological observations, only CAPEL treatment could effectively alleviate the upsurge of neutrophil infiltration via a significant 44% and 41% reduction in pancreatic MPO activity compared to the ORN group and CAPES-treated group, respectively. On the other hand, the CAPES-treated group failed to show any significant improvement in ornithine-induced neutrophil infiltration (Figure 7B).

From in vitro and in vivo results taken together, it can be concluded that encapsulation of CAPE in the optimal nanoliposomal formulation (CAPEL) significantly improves its effectiveness following oral administration in the alleviation of AP compared to an equivalent dose of oral CAPE suspension (CAPES). The superiority of CAPEL over CAPES in counteracting AP-associated oxidative stress, neutrophil infiltration, inflammation, apoptosis and energy depletion could be ascribed to the benefits associated with use of liposomes in the delivery of actives such as efficient cellular/tissue uptake, improved pharmacokinetic/pharmacodynamic profile, protection against enzymatic degradation and immunologic inactivation and probably altering the uptake mechanism or biodistribution of smaller particles compared to larger ones in the pancreas, thereby improving the drug efficacy.

It is worth noting that nanosystems were reported to decrease dose-related adverse effects associated with the encapsulated drugs. This was mainly attributed to difference in uptake mechanisms and/or biodistribution in body tissues of nano-sized particles, controlled release of the loaded drug, in addition to the overall improvement in the oral bioavailability of the encapsulated drug thus allowing dose reduction [73,74]. Therefore, the optimal nanoliposomal formulation of CAPE might reduce any adverse effects associated with the exposure to the free drug such as the reported propolis- and CAPE-induced allergy [75,76,77]. It has been shown that the most important allergic molecules in propolis are caffeic acid esters (especially caffeic acid phenyl ester), cinnamic acids and their aromatic esters [77,78]. Future studies will address the possible adverse effects associated with the oral administration of caffeic acid phenethyl ester (CAPE) and CAPE-loaded nanoliposomes (CAPE loaded-NL) in rats without induction of acute pancreatitis. Moreover, the reported protective role of adaptive cytoprotection in the pancreas will be investigated by combining ischemic preconditioning with L-ornithine-induced AP [65,79].

## 4. Conclusions

In this study, we demonstrated that an optimal nanoliposomal formulation loaded with CAPE could improve the efficacy of CAPE in the management of acute pancreatitis following oral administration which might lead to the clinical development of this effective approach. The liposomal nanoformulation of CAPE seems to provide a promising interventional approach for AP probably by altering the pharmacokinetic and pharmacodynamic profiles of CAPE. The currently developed nanoliposomal formulation enhanced the antioxidant, anti-inflammatory, and anti-apoptotic effects of CAPE probably through the modulation of NF-κβ and Nrf2 signaling. The study also highlights the great potential of the optimal nanoliposomal formulation to serve as a delivery platform for producing a variety of colloidal dispersions with high encapsulation efficiency and ultra-small size for the oral delivery of actives with poor oral bioavailability and sub-optimal efficacy. However, more in vivo studies are required to assess whether the results obtained using the animal model can be extrapolated to a clinical situation.

## Figures and Tables

**Table 1:** Mean (±SD) vesicle size (VS), polydispersity index (PDI), % entrapment efficiency (EE%) and zeta potential (ZP) of caffeic acid phenethyl ester (CAPE)-loaded nanoliposomes (CAPE-loaded-NL) prepared using Tween 80, sodium cholate (SC) or sodium deoxycholate (SDC) as edge activator (EA), (*n* = 3).

EA	VS (nm)	PDI	EE (%)	ZP (mV)
Tween 80	476 ± 9 ^b^*^,c^*	0.42 ± 0.04 ^b^*	77.1 ± 1.44 ^b^*^,c^*	−49.3 ± 3.68 ^b^*
SC	1211 ± 433 ^a^*^,c^*	0.90 ± 0.06 ^a^*^,c^*	93.5 ± 0.29 ^a^*^,c^*	−57.05 ± 0.78 ^a^*^,c^*
SDC	309 ± 54 ^a^*^,b^*	0.46 ± 0.12 ^b^*	86.9 ± 0.98 ^a^*^,b^*	−47.15 ± 0.78 ^b^*

* *p* < 0.05. ^a^ Versus Tween 80, ^b^ Versus SC, ^c^ Versus SDC.

**Table 2:** Effect of administering caffeic acid phenethyl ester (CAPE)-optimal liposomal formulation (CAPEL) on histological signs of pancreatic damage in rats with ornithine-induced acute pancreatitis.

	Control	ORN	ORN+CAPES	ORN+CAPEL
Acinar degenerative changes	0	3	1	0
Congested vasculature	0	1	0	0
Inflammatory cell infiltration	0	2	1	0

## Data Availability

Data is contained within the article.
